# PLC regulates spontaneous glutamate release triggered by extracellular calcium and readily releasable pool size in neocortical neurons

**DOI:** 10.3389/fncel.2023.1193485

**Published:** 2023-05-16

**Authors:** Maya G. Feldthouse, Nicholas P. Vyleta, Stephen M. Smith

**Affiliations:** ^1^Section of Pulmonary and Critical Care Medicine and Research and Development, VA Portland Health Care System, Portland, OR, United States; ^2^Division of Pulmonary and Critical Care Medicine, Oregon Health and Science University, Portland, OR, United States

**Keywords:** spontaneous release, PLC, minis, extracellular calcium, readily releasable pool, calcium-sensing receptor, GPCR

## Abstract

**Introduction:**

Dynamic physiological changes in brain extracellular calcium ([Ca^2+^]*_*o*_*) occur when high levels of neuronal activity lead to substantial Ca^2+^ entry *via* ion channels reducing local [Ca^2+^]*_*o*_*. Perturbations of the extracellular microenvironment that increase [Ca^2+^]*_*o*_* are commonly used to study how [Ca^2+^] regulates neuronal activity. At excitatory synapses, the Ca^2+^-sensing receptor (CaSR) and other G-protein coupled receptors link [Ca^2+^]*_*o*_* and spontaneous glutamate release. Phospholipase C (PLC) is activated by G-proteins and is hypothesized to mediate this process.

**Methods:**

Patch-clamping cultured neocortical neurons, we tested how spontaneous glutamate release was affected by [Ca^2+^]*_*o*_* and inhibition of PLC activity. We used hypertonic sucrose (HS) to evaluate the readily releasable pool (RRP) and test if it was affected by inhibition of PLC activity.

**Results:**

Spontaneous glutamate release substantially increased with [Ca^2+^]*_*o*_*, and inhibition of PLC activity, with U73122, abolished this effect. PLC-β1 is an abundant isoform in the neocortex, however, [Ca^2+^]*_*o*_*-dependent spontaneous release was unchanged in PLC-β1 null mutants (*PLC-β1^–/–^*). U73122 completely suppressed this response in *PLC-β1^–/–^* neurons, indicating that this residual [Ca^2+^]*_*o*_*–sensitivity may be mediated by other PLC isoforms. The RRP size was substantially reduced after incubation in U73122, but not U73343. Phorbol esters increased RRP size after PLC inhibition.

**Discussion:**

Together these data point to a strong role for PLC in mediating changes in spontaneous release elicited by [Ca^2+^]*_*o*_* and other extracellular cues, possibly by modifying the size of the RRP.

## Introduction

Spontaneous transmission, occurring in the absence of an action potential, comprises a substantial fraction of neurotransmitter release and is key to early synapse formation (Andreae et al., 2012) and maintenance of synaptic strength (McKinney et al., 1999). Spontaneous and action potential-evoked neurotransmitter release arise from distinct vesicle pools (Sara et al., 2005; Fredj and Burrone, 2009; Melom et al., 2013) and appear to be regulated by different mechanisms (Phillips et al., 2008; Ramirez and Kavalali, 2011; Vyleta and Smith, 2011), however, both forms of release are proportional to extracellular calcium concentration ([Ca^2+^]_*o*_) (Neher and Sakaba, 2008; Vyleta and Smith, 2011). While action potential-triggered calcium entry via voltage-activated calcium channels (VACCs) mediates evoked release (Katz, 1969; Llinás et al., 1976; Wheeler et al., 1994), many questions remain unanswered about how extracellular calcium regulates spontaneous release. At inhibitory synapses, spontaneous release is triggered by calcium influx via VACCs (Goswami et al., 2012; Williams et al., 2012) but this is not the case at excitatory synapses (Abenavoli et al., 2002; Vyleta and Smith, 2011; Tsintsadze et al., 2017; Babiec and O’Dell, 2018; Courtney et al., 2018). Therefore, we sought to identify other mechanisms by which [Ca^2+^]_*o*_ affects the frequency of spontaneous release at excitatory synapses.

Many G-protein coupled receptors (GPCRs) regulate spontaneous glutamate release in neurons. Stimulation of metabotropic glutamate receptors (mGluR) (Simkus and Stricker, 2002; Xie et al., 2017), the calcium sensing receptor (CaSR) (Vyleta and Smith, 2011), and the α1-adrenoceptor (Choy et al., 2018) all lead to increases in spontaneous glutamate release which manifest as miniature excitatory postsynaptic currents (mEPSCs). Moreover the [Ca^2+^]_*o*_ dependence of spontaneous release can be altered by specific GPCRs such as mGluR1 (Kubo et al., 1998; Tabata et al., 2002), CB1 (Yamasaki et al., 2006), CaSR (Vyleta and Smith, 2011), and GABA_*B*_ receptor (Yamasaki et al., 2006). Phospholipase C (PLC) is well positioned as a target molecule linking extracellular calcium and spontaneous release since it sits downstream to many of these GPCRs and, once activated, catalyzes the hydrolysis of phosphatidylinositol-(4,5)-bisphosphate (PIP_2_) to inositol triphosphate (IP_3_) and diacylglycerol (DAG) (Berridge and Irvine, 1984; Rhee, 2001; Horowitz et al., 2005). Exogenous DAG analogs increase the rate of spontaneous exocytosis (Wierda et al., 2007; Lou et al., 2008) and the size of the readily releasable pool (RRP) of vesicles in central neurons (Stevens and Sullivan, 1998) while PIP_2_ has been identified as a key molecule in regulation of vesicle endo- (Liu et al., 2009) and exocytosis (Bai et al., 2004; Groffen et al., 2010). We hypothesized that PLC activation by calcium-detecting GPCRs mediates [Ca^2+^]_*o*_-dependent spontaneous glutamate release.

Here, we interrogated how spontaneous synaptic transmission is impacted by pharmacological and genetic modulation of PLC. Our data show that basal and [Ca^2+^]_*o*_-dependent spontaneous release are dependent on PLC activity. Moreover, PLC inhibition decreased the size of the RRP as measured with hypertonic sucrose (HS) stimulation. These findings confirm a strong role for PLC in regulation of vesicle pools and their release, and imply a GPCR mechanism mediating the enhanced frequency of spontaneous release in response to [Ca^2+^]_*o*_.

## Materials and methods

### Cell culture preparation

Neocortical neurons were isolated from both male and female P1-2 mouse pups as reported previously (Phillips et al., 2008). All animal procedures were approved by VAPORHCS IACUC in accordance with the U.S. Public Health Service Policy on Humane Care and Use of Laboratory Animals and the NIH Guide for the Care and Use of Laboratory Animals. Animals were deeply anesthetized with isoflurane before decapitation. The brain was extracted and cortices were dissected in cold Mg^2+^- and Ca^2+^-free Hanks’ balanced salt solution (Corning) plus 20% fetal bovine serum (FBS). Cortices were then incubated in trypsin (5 mg/mL) and DNAse (0.1 mg/mL) and then dissociated with a heat polished pipette in a high magnesium solution. Dispersed cells were cultured in minimal essential medium plus 5% FBS (MEM+) on glass coverslips that had been pre-coated with dilute Matrigel. ARAC (4 μM) was added 48–72 h after plating to limit glial division, then diluted two-fold with MEM + (2 μM final concentration) 72 h later. Cells were used after ≥ 14 days in culture.

### Animals

The vast majority of experiments utilized cultures derived using a C57BL/6J and 129/SvJ mouse strain. We also used PLC-β1 null-mutant (*PLC-*β*1^–/–^*) mice, kindly provided by Dr. Hee-Sup Shin (KIST). The PLC-β1*^–/–^* mice were generated by a gene targeting method as described previously (Kim et al., 1997). Briefly, the PLC-β1 was disrupted by homologous recombination and transfected into embryonic stem cells. In our hands, the homozygous mutation was lethal and we were unable to generate any PLC-β1*^–/–^* pups. This differed from other studies who reported a reduced life expectancy compared to wild-type due to seizures and sudden death (Kim et al., 1997). The cause for the higher lethality we observed was unidentified. In an attempt to improve survival, we changed the underlying strain by back-crossing heterozygotes (strain C57BL/6J) with wild-type (WT) C57BL/6J and 129/SvJ mice for seven generations. This back-cross substantially increased the survival of the PLC-β1*^–/–^* mice. Then, mice heterozygous for the mutation\were crossed to produce animals for PLC-β1*^–/–^* and WT (PLC-β1^+/+^) cultures. The mutation consists of a PGK-neomycin cassette inserted into the exon encoding amino acids 50–82 of the protein for gene disruption. To genotype, DNA from mouse tail samples was extracted by treatment with 25 mM NaOH and 0.2 mM EDTA at 98°C for 30–50 min. Forty mM Tris buffer (20 mM final), pH 5.5, was added and the mixture centrifuged. The DNA-containing supernatant was used for polymerase chain reaction (PCR). PCR was performed using three primers: F1: 5′-CAAGTTAAGTCCGGCAAACACC-3′, B1: 5′-ACCTTGGGAGCTT-TGGCGTG-3′, and PGK22: 5′-CTGACTAGGGGAGGAGTAGAAG-3′. The PCR products were run on 1% agarose gels. Previous microarray experiments of 2 week old cultures using the GeneChip Clariom S Mouse Array (Affymetrix/Applied Biosystems) (Lindner et al., 2022) were analyzed using Affymetrix Expression Console software ver.1.4.1.46. The microarray data are available at NCBI GEO (GSE218028).

### Electrophysiological recordings

**FIGURE 1:**
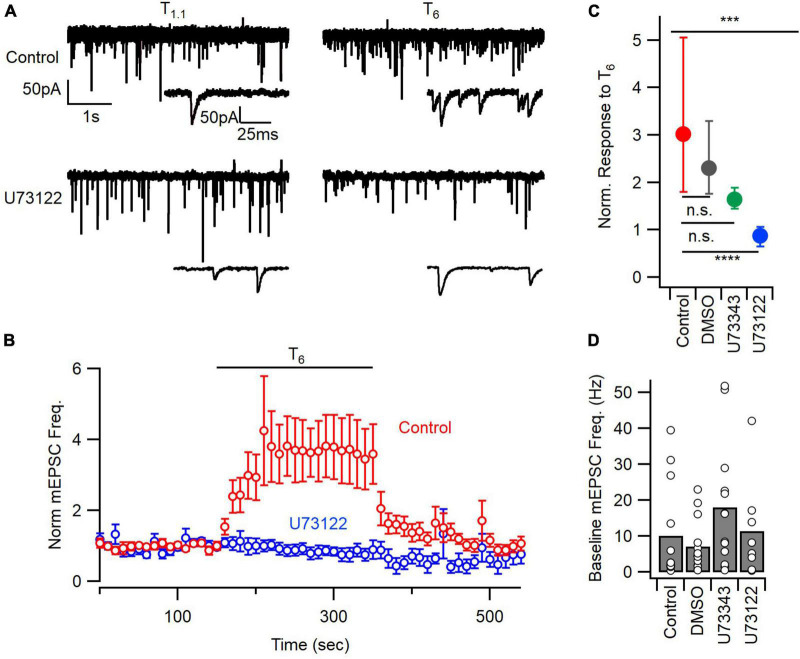
PLC inhibition blocks the mEPSC frequency response to extracellular calcium. **(A)** Sample traces of mEPSCs before (T_1_._1_) and after application of 6 mM calcium (T_6_), from control and U73122 (5 μM)-treated neurons. **(B)** Diary plot depicting the average mEPSC frequency pre-, during, and post- a 200 s T_6_ application, normalized to the baseline frequency in T_1_._1_. Error bars indicate ± SEM. **(C)** The median (solid circles) normalized mEPSC frequency in T_6_ was decreased by U73122. n.s., non-significant; ***p < 0.001; ****p < 0.0001. Top asterisks represent initial testing significance result, and those below show the significance of *post hoc* comparisons. **(D)** Baseline mEPSC frequency in 1.1 mM [Ca^2+^]_o_ was not different between groups by ANOVA. Bars represent means and individual data points are shown as open circles.

Cells were visualized with an Olympus IX70 or Zeiss IM35 × 40 inverted microscope. Recordings were made in whole-cell voltage clamp mode using a HEKA 9 or 10/2 amplifier. Holding potential was −70 mV, corrected for experimentally-derived liquid-junction potentials and cells were perfused with extracellular solution containing (in mM) 150 NaCl, 4 KCl, 1.1 CaCl_2_, 1.1 MgCl_2_, 10 HEPES, 10 glucose, pH 7.35. In experiments where [Ca^2+^]_*o*_ was manipulated (Figures 1, 3), the concentration of [Mg^2+^]_*o*_ was decreased to 0.5 mM. Recordings of mEPSCs and hypertonic sucrose-induced currents were made in the presence of tetrodotoxin (TTX, 1 μM) and bicuculline (Bic, 10 μM) to block voltage-gated sodium channel currents and GABA-activated currents, respectively. Both potassium gluconate and cesium methane-sulfonate pipette solutions were used. Potassium gluconate solution consisted of (in mM) 113 K^+^ gluconate, 9 EGTA, 10 HEPES, 4 MgCl_2_, 1 CaCl_2_, 4 Na_2_ATP, 0.3 diphosphate-GTP, 14 creatine phosphate, pH 7.2. Cesium methane-sulfonate solution consisted of (in mM) 108 Cs methanesulfonate, 9 EGTA, 10 HEPES, 4 MgCl_2_, 1 CaCl_2_, 4 Na_2_ATP, 0.3 diphosphate-GTP, 14 creatine phosphate, pH 7.2. Patch electrodes had resistances of 2–7 MΩ. Miniature EPSCs were filtered at 1 kHz and digitized at 10 kHz. For recording of HS-induced currents (Figures 4–6), currents were filtered at 3 kHz and sampled at 20 kHz. Series resistance (Rs) was monitored, and recordings were discarded if Rs changed significantly during the course of a recording. Rs was compensated by up to 70%.

**FIGURE 2:**
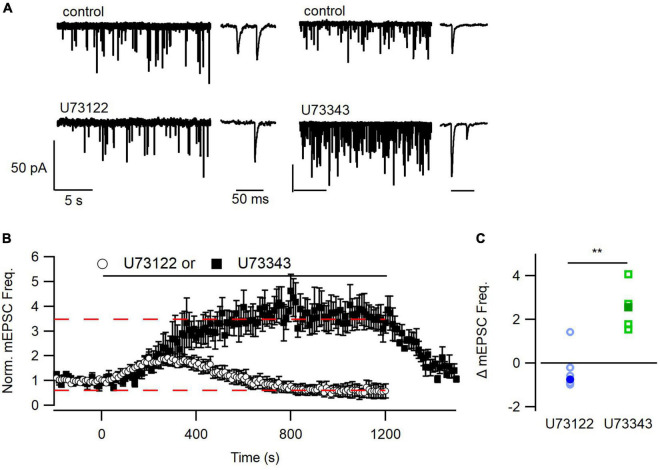
Time course of inhibition of mEPSC frequency by U73122. **(A)** Representative traces from before and after application of U73122 or U73343. **(B)** Diary plots depicting the group average pre-, during, and post-application of either U73122 (5 μM) or inactive analog U73343 (5 μM), normalized to the pre-application baseline. U73343 produced a robust potentiation of spontaneous release that was completely reversible (dashed line at 3.46). U73122 on average produced complex effects including an early increase and late oscillation to below baseline levels (dashed line at 0.596). **(C)** U73122 reduced the median (solid markers) treatment-induced change in mEPSC frequency values, relative to baseline, compared to U73343. Values from individual experiments are shown as open markers and averages as solid markers here and in following histograms. **p < 0.01.

**FIGURE 3:**
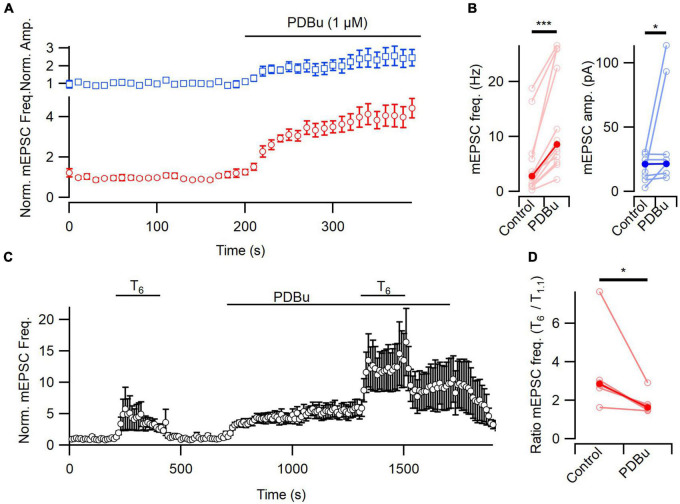
[Ca^2+^]_*o*_-sensitivity of spontaneous release attenuated by PDBu. **(A)** Diary plots of the frequency and amplitude of mEPSCs during the wash on of PDBu (1 μM) in T_1_._1_. **(B)** The absolute frequency and amplitude of mEPSCs increased after PDBu application, compared to baseline (red and blue, respectively). **(C)** A diary plot showing the average effect (*n* = 6) of elevating [Ca^2+^]_o_ from 1.1 to 6 mM (T_6_) on mEPSC frequency before and after PDBu application, normalized to baseline. PDBu does not occlude the enhancement of spontaneous release by extracellular Ca^2+^. **(D)** The [Ca^2+^]_*o*_-dependent increase in mEPSC frequency was attenuated by PDBu in this plot of the ratio of mEPSC frequency in T_6_ and T_1_._1_ (*n* = 6). Average frequency measured over the last 80 s of each solution application. All error bars represent ± SEM. *p < 0.005; ***p < 0.001.

**FIGURE 4:**
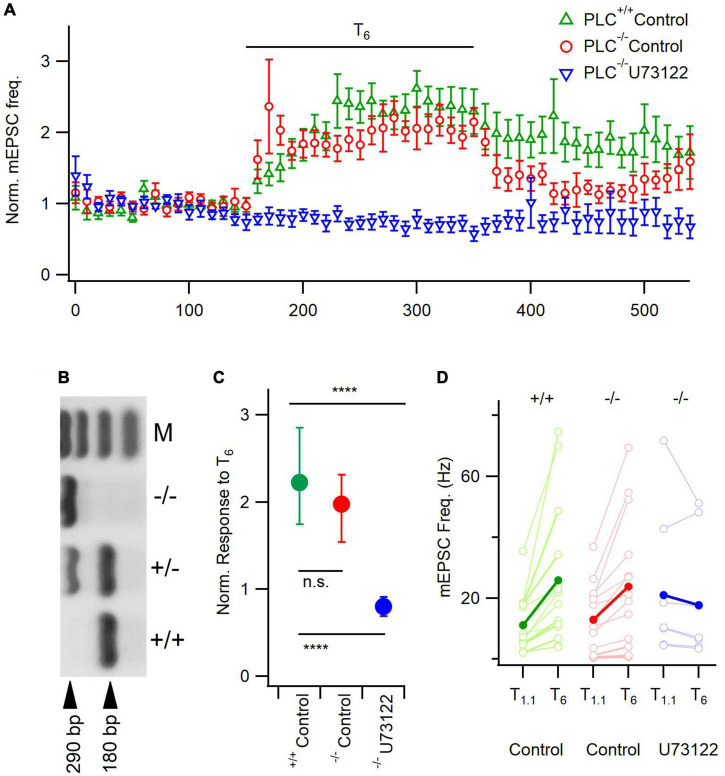
U73122 inhibition of mEPSCs is unaffected by PLC-β1 deletion. **(A)** Diary plot depicting the average mEPSC frequency ± SEM pre-, during, and post- a 200 s T_6_ application, normalized to the frequency in T_1_._1_. **(B)** Gel confirming *PLC-*β*1^–/–^* animals by the presence of a single band at 290 bp. M is a 100 bp ladder, showing bands 100–400 bp. **(C)** The median response size to T_6_, normalized to treatment baseline, is not different between *PLC-*β*1^–/–^* animals and *PLC-*β*1*^+^*^/^*^+^ litter mate controls, however, U73122 treatment still blocks this response in *PLC-*β*1^–/–^* neurons. Error bars indicate first and third quartile values. n.s., non-significant; ****p < 0.0001. Top asterisks represent initial testing significance result, and those below show the significance of *post hoc* comparisons. **(D)** Absolute mEPSC frequency response demonstrates similar median (solid circles) basal frequencies in T_1_._1_ between groups. Individual neuron data (open circles) illustrate no effect of basal rates on response to T_6_.

**FIGURE 5:**
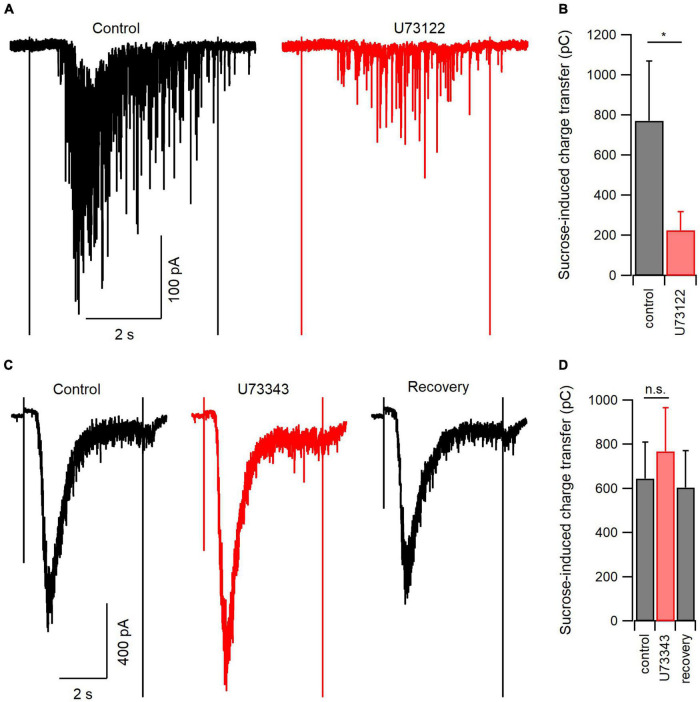
PLC blockade reduces the size of the readily releasable pool of synaptic vesicles. **(A)** Representative hypertonic sucrose-induced currents (H.S.; 500 mM) directly before (control, black) and at the end of a 20-min application of U73122 (5 μM, red). **(B)** Summary data of the experiments represented in panel **(A)**. On average, U73122 inhibited total sucrose-induced charge transfer by 73%. **(C)** Representative H.S.-induced currents before (control, black), at the end of a 20-min application of U73343 (5 μM, red), or after 5 min of washout (recovery, black). **(D)** Summary data of the experiments represented in panel **(C)**. There was a trend toward U73343 reversible increasing sucrose-induced charge transfer (*n* = 7 for control and U73343 comparison, 4 for recovery). n.s., non-significant; *p < 0.05.

**FIGURE 6:**
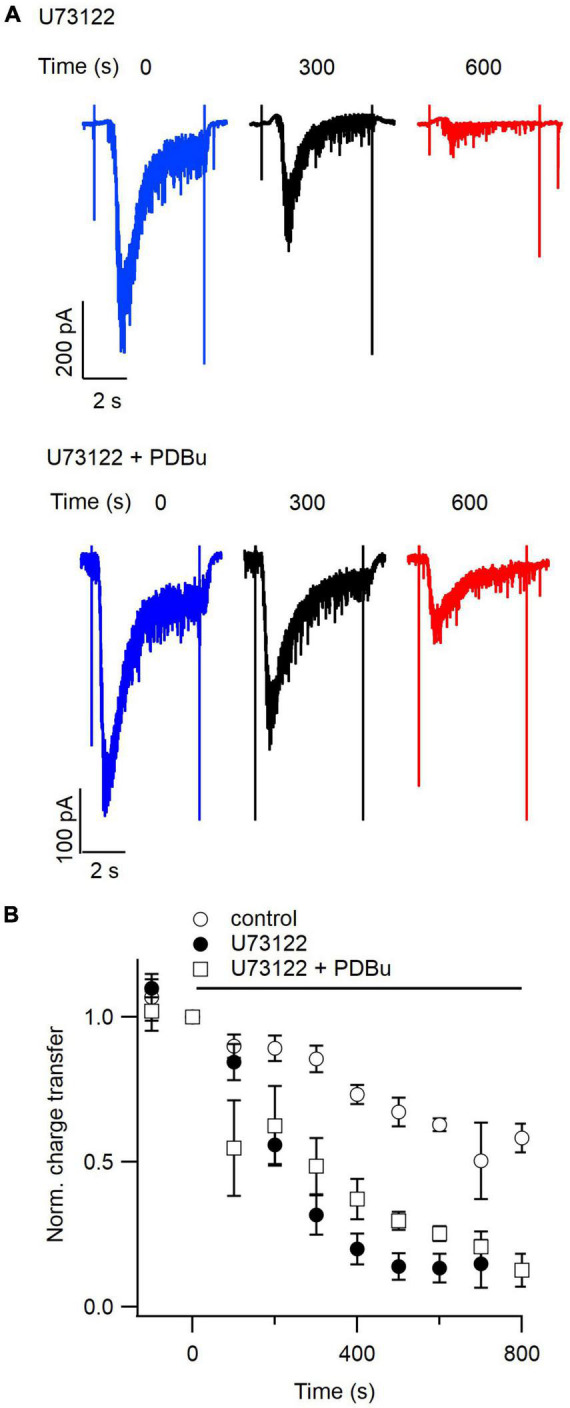
Phorbol esters partially prevent inhibition of the readily releasable pool by U73122. **(A)** Representative hypertonic sucrose-induced currents evoked every 100 s. The traces shown represent currents elicited just before (blue), 300 s after (black), or 600 s after (red) simultaneous application of U73122 (5 μM, upper) and U73122 plus PDBu (1 μM, lower). **(B)** Average normalized diary plots of total sucrose-induced charge transfer versus time for currents evoked with a 100 s inter-stimulus interval in control conditions (open circles), during application of U73122 alone (closed circles), or during simultaneous application of U73122 and PDBu (open squares). Data are normalized to values at time zero. Error bars indicate ± SEM.

### Solution and drug application

For recordings of mEPSCs, solutions were bath-applied constantly through a perfusion pipette placed ∼1 mm from the patch pipette tip. The perfusion pipette was connected to a manifold (Warner Instruments) and stopcocks used to switch solutions. Local solution equilibration occurred in substantially less than 10 s as measured by open-tip conductance changes. U73122 and U73343 stocks (5 mM) were prepared in DMSO and diluted 1:1000 in Tyrode’s for experiments which resulted in final drug concentrations of 5 μM and a final vehicle concentration of 0.1% in the recording solutions, which had no apparent effect in control experiments. Preparation of stocks required warming to 30°C in water bath then agitation using a sonicator (20 kHz for 10 × 10 s burst, FB150, Fisher Scientific, Hampton, NH, USA).

To measure the RRP, we evoked fusion of the vesicles with a hypertonic extracellular solution (Tyrode plus 500 mM sucrose) applied for 5 s using a custom-built Piezo-driven perfusion system described previously (Vyleta and Smith, 2008) and recorded the corresponding currents in the post-synaptic cell. Briefly, a piece of double-barreled theta glass was used to perfuse both control and hypertonic sucrose solutions simultaneously. A whole-cell recording was made from a neuron being bathed only by the control solution. Upon stimulation by a high-voltage stimulus isolator (World Precision Instruments, Sarasota, FL, USA, product # A365D), a Piezo bimorph (Piezo Systems, Inc., product # T234-A4CL-203X) moved the tip of the theta glass horizontally so that the recording was then bathed by extracellular solution containing hypertonic sucrose. Each barrel of the theta glass contained two small capillaries delivering solutions by gravity to the recording chamber.

### Analysis

Miniature EPSC frequency was measured in 10 s bins and normalized to the average mEPSC frequency during the preceding ≥ 100 s of recording in 1.1 mM [Ca^2+^]_*o*_ and 0.5 or 1.1 mM [Mg^2+^]_*o*_ (details in legends). The response to elevated [Ca^2+^]_*o*_ and other interventions reflects the average relative frequency measured over the last 80–100 s of the 6 mM calcium application, reflecting the steady state response. Analysis was performed using IgorPro (Wavemetrics, Lake Oswego, OR, USA) software. Single spontaneous release events were detected using a template matching method (Clements and Bekkers, 1997) using routines written by Dr. H. Taschenberger (López-Murcia et al., 2019). Data were acquired on a PIII computer and analyzed with IgorPro (Wavemetrics, Lake Oswego, OR, USA) software. Miniature EPSCs were identified using an 8 ms current template that comprised of a baseline (0.4 ms), a rising phase (tau 0.4 ms), and a decaying phase (tau 2.5 ms). The template was occasionally altered to better fit the shape of mEPSCs in an individual recording. IgorPro software analyzed the compliance of the raw current data to the template and gave a criterion score based on how well each segment of the data matched the template. A criterion threshold was set such that recording noise scored below the threshold, and obvious mEPSCs scored above threshold. All criterion scores at or above that value were identified as mEPSCs.

Hypertonic sucrose-induced charge transfer, representing the RRP, was calculated as the integral of the total currents induced over 5 s of sucrose application. The duration of sucrose application was clearly identified by stimulus artifacts in the recordings (Figures 5, 6). In some experiments, currents did not show clear transient and steady-state phases. Thus total current elicited by HS was integrated in all experiments, without subtracting off a steady-state component, and used to estimate the RRP (Stevens and Sullivan, 1998). Curve fitting was performed using IgorPro (Wavemetrics, Lake Oswego, OR, USA).

### Statistics

Statistical significance was determined using parametric or non-parametric testing using ANOVA or Kruskal–Wallis tests with Dunn’s multiple comparisons tests or Student’s *t*-test or Mann-Whitney test as appropriate (Microsoft EXCEL, Richmond, WA; Graphpad Prism). Non-Gaussian distribution data are reported displaying the median. Normally distributed data were reported as mean ± SEM (text and legends). *P*-values < 0.05 were considered significant and cut-offs were denoted in Figures with the following symbols (*p*-values < 0.05, 0.01, 0.001, and 0.0001 were indicated with *, ^**^, ^***^, and ^****^, respectively and n.s. used to identify non-significant *p*-values).

## Results

### Extracellular [Ca^2+^] dependence of spontaneous release requires PLC activity

A number of GPCRs have been implicated in the regulation of spontaneous release by [Ca^2+^]_*o*_ (Kubo et al., 1998; Tabata et al., 2002; Yamasaki et al., 2006; Vyleta and Smith, 2011). We tested if PLC, downstream of GPCRs, was contributing to the enhancement of spontaneous glutamate release by elevated [Ca^2+^]_*o*_ using the PLC inhibitor, U73122 (Smith et al., 1990; Horowitz et al., 2005). Whole-cell patch clamp recordings were made from neocortical neurons voltage-clamped at −70 mV in the presence of tetrodotoxin (TTX, 1 μM) and bicuculline methiodide (10 μM). We measured mEPSC frequency at physiological [Ca^2+^]_*o*_ (Tyrode with 1.1 mM [Ca^2+^]_*o*_, T_1_._1_) and then following a step to 6.0 mM [Ca^2+^]_*o*_ (Tyrode with 6 mM [Ca^2+^]_*o*_, T_6_) in untreated controls, or following a 20- to 50-min incubation with U73122 (5 μM), the vehicle DMSO, or the inactive analog, U73343 (5 μM; Figure 1). Exemplar traces show that the mEPSC frequency increased robustly in response to T_6_ but that this increase was attenuated by U73122 (Figure 1A). Similarly, the time course of the response to T_6_, which occurred over multiple 10 s of seconds consistent with earlier reports (Vyleta and Smith, 2011), was absent following U73122 treatment (Figure 1B). Key controls included testing if the response to T_6_ was affected by the vehicle, DMSO, or the inactive analog, U73343. In a comparison of the normalized response to T_6_, the medians were different (Figures 1C; *P* = 0.0002 by Kruskal–Wallis test). The control group increased to 3.02 of baseline which was substantially higher than the 0.88 times baseline observed after U73122 (5 μM) treatment (*P* = 0.0001 by Dunn’s multiple comparisons). The control responses to T_6_ were unaffected by treatment with DMSO (median 2.3 times baseline, *n* = 14, *P* > 0.9999) or U73343 (median 1.6 times baseline, *n* = 13, *P* = 0.2058, Dunn’s; Figure 1C). Taken together these data indicate that [Ca^2+^]_*o*_ stimulation of spontaneous release of glutamate is dependent on the activation of PLC.

Ca^2+^-sensing receptor detects changes in [Ca^2+^]_*o*_ and is estimated to regulate ∼30% of basal spontaneous glutamate release in neocortical neurons (Vyleta and Smith, 2011). We hypothesized that PLC may also determine the mEPSC frequency in the presence of T_1_._1_. However, basal mEPSC frequency was not impacted by U73122, U73343, or DMSO (*p* = 0.33, Kruskal–Wallis; Figure 1D). The large population variance in basal mEPSC frequency increases the likelihood that differences may not be detected (Figure 1D). Consequently, we examined the time-course of U73122 and U73343 effects on basal mEPSC frequency within single cells. Using this approach, we observed that the response to U73122 was biphasic and consisted of an initial increase then subsequent decrease in the average normalized mEPSC frequency (Figures 2A, B). At steady state, U73122 decreased mEPSC frequency by 40 ± 28% (*n* = 8, red line Figure 2B). The response to U73443 was monophasic with spontaneous glutamate release increasing by 246 ± 44% (*n* = 5) following U73443 application. The median steady state mEPSC frequency changed to 74% and 355% of baseline for U73122 and U73343, respectively, (*P* = 0.0016, Mann–Whitney, Figures 2B, C). This difference probably indicates that a fraction of basal spontaneous glutamate release is PLC-dependent. However, the similarity of the initial increase in mEPSC frequency caused by both U73122 and U73343 indicates these agents presumably have off-target effects.

### DAG affects baseline mEPSC response, and attenuates [Ca^2+^]_*o*_ effect

Phospholipase C modulates signaling by increasing IP_3_ and DAG (Berridge and Irvine, 1984). Stimulation of spontaneous release by increasing [Ca^2+^]_*o*_ was reduced by buffering intracellular [Ca^2+^] ([Ca^2+^]_*i*_) at inhibitory but not excitatory synapses (Vyleta and Smith, 2011; Williams et al., 2012). This finding indicates that IP_3_-induced rises in [Ca^2+^]_*i*_ were an unlikely cause for the change in mEPSC frequency to T_6_ (Figure 1). Therefore, we next tested if PLC activation by presynaptic GPCRs might enhance spontaneous release rate by increasing levels of DAG. Phorbol esters are synthetic DAG analogs that enhance spontaneous release in central neurons (Lou et al., 2008). Consistent with this, phorbol 12,13-dibutyrate (PDBu, 1 μM) produced a robust enhancement of spontaneous release in the presence of T_1_._1_ following a 200 s application (4.16 ± 0.45 times baseline, *n* = 12, *P* < 0.001; Figures 3A, B). This result is consistent with DAG increasing spontaneous release of glutamate. Additionally, there was a modest increase of mEPSC amplitude after PDBu treatment (mean 2.11 ± 0.54 times baseline, but similar median values, *P* = 0.039 by Wilcoxon test; Figures 3A, B), which is not consistent with previous data (Lou et al., 2008). If elevation of [Ca^2+^]_*o*_ enhances the rate of spontaneous neurotransmission by producing DAG, then exogenously applied DAG analogs may impair the effect of elevation of [Ca^2+^]_*o*_ on spontaneous release. To examine the interaction between a high intracellular concentration of phorbol ester and cell’s ability to respond to the addition of elevated [Ca^2+^]_*o*_, we applied PDBu and subsequently added T_6_. T_6_ increased normalized mEPSC frequency to 3.52 ± 1.03 of baseline and PDBu increased it to 5.49 ± 1.12 and 12.64 ± 3.68 of baseline in T1.1 and T6, respectively (Figure 3C, *n* = 6). The median ratio of mEPSC frequency in T_6_ to T_1_._1_ decreased significantly from 2.8 to 1.6 following PDBu treatment (*P* = 0.031) indicating that the phorbol ester reduced the sensitivity of spontaneous release to increases in [Ca^2+^]_*o*_.

### PLC subtype

Some studies indicated that PLC-β1 is the most abundant PLC isoform in the neocortex (Ross et al., 1989; Lim et al., 2022). Therefore, we tested if PLC-β1 mediated [Ca^2+^]_*o*_-dependent spontaneous release of glutamate by comparing the sensitivity of mEPSC frequency between neurons deficient of PLC-β1 (*PLC-*β*1^–/–^*) with littermate control neurons (*PLC-*β*1*^+^*^/^*^+^) and the impact of U73122 in *PLC-*β*1^–/–^* cultures (Figure 4). These genotypes were confirmed using PCR (Figure 4B). There were significant differences between the response to T_6_ across the three groups (Kruskal-Wallis, *P* < 0.0001). In *PLC-*β*1^–/–^* neurons and littermate control neurons (*PLC-*β*1*^+^*^/^*^+^), mEPSC frequency increased similarly following application of T_6_ (median = 2.0 and 2.2 times baseline, respectively, *n* = 14 for both groups, *p* = 0.94, Dunn’s; Figures 4A, C). However, the response to T_6_ was completely suppressed in U73122 pre-treated *PLC-*β*1^–/–^* neurons (median = 0.8 times baseline, *p* < 0.0001, Dunn’s; Figures 3A, C). These data indicate that PLC-β1 may not be contributing to the [Ca^2+^]_*o*_-dependent spontaneous release of glutamate in wild-type neurons or there may be upregulation of compensatory pathways (Namkung et al., 1998). Alternatively, more recent data has shown that other PLC isoforms may have a substantial presence in the neocortex (Lindner et al., 2022) possibly facilitating these [Ca^2+^]_*o*_-dependent changes in spontaneous release in *PLC-*β*1^–/–^* neurons.

### PLC activity maintains the size of the readily releasable pool of synaptic vesicles

Earlier experiments indicated that basal spontaneous release of glutamate was inhibited by U73122, indicating that PLC signaling may contribute to vesicle turnover and availability. The size of the RRP is one determinant of the probability of neurotransmission. To test if PLC regulates RRP size we elicited exocytosis by applying HS solution and recorded excitatory postsynaptic currents (EPSCs) in voltage-clamp (Rosenmund and Stevens, 1996). Hypertonic sucrose (Tyrode plus 500 mM sucrose) evoked large inward currents that were inhibited by a 20-min application of U73122 (Figure 5A). On average, the total HS-induced charge transfer was 772 ± 299 pC before and 225 ± 93 pC after U73122 (*n* = 7, *P* = 0.048, paired *t*-test; Figure 5B). This inhibition was not reversible during our experiments, as reported by others (Jin et al., 1994). We next confirmed this inhibition of synaptic vesicle fusion was specific to PLC blockade by measuring the effect of U73343 on sucrose-induced currents. A 20-min application of U73343 produced an apparent increase in sucrose-induced currents that was reversible (Figures 5C, D), however, this trend failed to reach statistical significance. On average, total sucrose induced charge transfer was 645 ± 165 pC before and 769 ± 198 pC after U73343 (*n* = 7, *P* = 0.061, paired *t*-test; Figure 5D). Taken together, these results indicate inhibition of PLC decreases the size of the synaptic vesicle pool available to fuse in response to hypertonic solution, and further suggests that at baseline, PLC regulates the size of the RRP in excitatory neocortical neurons.

### DAG analogs only partially rescue inhibition of RRP by PLC blockade

Phorbol esters increase both the size and recovery rate of the RRP of synaptic vesicles evoked with hypertonic sucrose solution (Stevens and Sullivan, 1998). We hypothesized that if U73122 decreases RRP size by decreasing DAG levels in the presynaptic membrane, then the inhibition of sucrose-induced currents by PLC blockade should be rescued by exogenous phorbol ester. We evoked hypertonic sucrose-induced currents every 100 s while simultaneously applying U73122 and PDBu. Representative traces from one recording of sucrose-induced EPSCs at 300 and 600 s after application of U73122 illustrate U73122 still inhibits neurotransmitter release in the presence of PDBu (Figure 6A). The average normalized diary plots of total sucrose-induced charge transfer versus time suggest that phorbol ester application may slow the depletion of the RRP seen under PLC blockade (Figure 6B). A comparison of total sucrose-induced charge transfer between 500 and 600 s after onset of U73122 application indicates that inhibition of RRP is less complete in the presence of PDBu. Average total charge transfer was 0.14 ± 0.03 and 0.28 ± 0.02 of control for U73122 alone and U73122 + PDBu (*n* = 14 and 6 data points, respectively, *P* = 0.02), however, the two curves converge by 700 s of U73122 application (Figure 6B). Average time constants for inhibition of sucrose-induced currents 220 ± 44 and 349 ± 86 s for U73122 alone and U73122 + PDBu respectively, suggesting that PDBu slows RRP inhibition, however this difference was not statistically significant (*n* = 8 and 3, respectively, *p* = 0.18 test). Taken together, these results suggest that inhibition of neurotransmitter release by PLC blockade may be in part due to a consequent decrease in DAG levels in the presynaptic membrane.

## Discussion

Here we demonstrate the enhancement of mEPSC frequency by elevation of extracellular calcium is dependent on intact PLC signaling. We also found PLC regulates the RRP, possibly via a mechanism involved in the replenishment of these vesicles. Both effects appeared to be moderately affected by PDBu, implying a role for DAG in mEPSC frequency and RRP regulation.

Rises in [Ca^2+^]_*o*_ result in increases in mEPSC frequency, however, this is independent of increases in [Ca^2+^]_*i*_ (Vyleta and Smith, 2011). Previously, we found that application of other agonists of the CaSR, a GPCR know to be present at nerve terminals (Chen et al., 2010), increased mEPSC frequency (Vyleta and Smith, 2011). We hypothesized that this effect is transduced by a GPCR, as a number of studies have found that stimulation of GPCRs increases mEPSC frequency (Jennings et al., 2003; Marinelli et al., 2003; Pi et al., 2005; Sikand and Premkumar, 2007). The G_*q*_/PLC pathway was therefore a candidate mediator between [Ca^2+^]_*o*_ and spontaneous release frequency.

We asked whether the enhancement of spontaneous release by [Ca^2+^]_*o*_ required PLC activity. Our finding that treatment with U73122, but not U73343, completely blocked the effects of elevated [Ca^2+^]_*o*_ on mEPSC frequency support the proposal that PLC is an important component of this signaling pathway. However, because U73122 and U73343 both cause an increase in basal mEPSC with similar kinetics (Figure 2B) this indicates that there are also concomitant PLC-independent effects of these agents. Explanations for the rapid increases in mEPSC frequency include a transient increase in [Ca^2+^]_*i*_, similar to that reported in rabbit gastric mucosa (Muto et al., 1997), in digitonin-permeabilized rat and mouse pancreatic acinar cells (Mogami et al., 1997), in Chinese hamster ovary cells (Karashima et al., 2008), and in human embryonic kidney cells (Horowitz et al., 2005). The kinetics of the reported changes in [Ca^2+^]_*i*_ occurred with a similar time course to the increase in mEPSC frequency after application of U73122 and U73343 (Figure 2B). We hypothesize that these transient changes in [Ca^2+^]_*i*_ drive an increase in vesicle fusion, which would eventually decrease as PLC inhibition by U73122 prevented further spontaneous release by decreasing the RRP. The inhibition of PLC by U73122, in human polymorphonuclear neutrophils and platelets (Bleasdale et al., 1990; Smith et al., 1990) and NG108-15 cells (Jin et al., 1994) developed more slowly over several minutes, like mEPSC inhibition by U73122 (Figure 2B). The reported time course of action of U73122 and the difference between the actions of U73122 and U73433, support the proposal that PLC inhibition decreases spontaneous release of glutamate. However, as emphasized in earlier reports the action of U73122 is complex and irreversible making it a difficult compound to use (Horowitz et al., 2005). We have not tested if PLC alkylation underlies the irreversibility of the inactivation by U73122 as has been proposed (Horowitz et al., 2005).

Phorbol 12,13-dibutyrate increased basal mEPSC frequency and decreased [Ca^2+^]_*o*_-enhancement of spontaneous release (Figure 3) consistent with a role for DAG-dependent regulation of spontaneous release. However, additional experiments are required to confirm that [Ca^2+^]_o_ and PDBu stimulate mEPSCs using the same mechanism.

We observed that PLC-β1 deletion did not attenuate the enhancement of mEPSC frequency by elevated [Ca^2+^]_o_. This result indicates that PLC-β1 may not be contributing to the [Ca^2+^]_*o*_-dependent spontaneous release of glutamate in wild-type neurons or alternatively that there may be upregulation of compensatory pathways in the null-mutant (Namkung et al., 1998). This would contrast with calcium-dependent GPCR-mediated modulation of evoked neurotransmission in hippocampal neurons (Hashimotodani et al., 2005), where the PLC-β1 isoform is essential, supporting the thesis that the roles of PLC isoforms are location-dependent within the brain (Rusciano et al., 2020). Since the *PLC-*β*1^–/–^* mouse has successfully been used to identify PLC-β1-sensitive signaling pathways in other parts of the brain (Hashimotodani et al., 2005) we suspect upregulation of other pathways is a less likely explanation. Other reports indicated that PLC-β1 is the most prevalent isoform in the cortex (Ross et al., 1989) and provided the rationale to use the PLC-β1 null mutant. However, recent gene profiling data (Lindner et al., 2022) has shown that expression levels of PLC-β3 and PLC-β4 are the same as those of PLC-β1 in comparable neocortical cultures to those used here (NCBI GEO, GSE218028). The relatively high levels of PLC-β3 and PLC-β4 expression in neocortical cultures provides a potential explanation why the *PLC-*β*1^–/–^* neurons demonstrated [Ca^2+^]_*o*_-dependent spontaneous glutamate release comparable to *PLC-*β*1*^+^*^/^*^+^ animals. Our finding that U73122 blocked the response to high [Ca^2+^]_o_ in wild-type and PLC-β1 null mutant neurons suggests that other PLC isoforms are involved in mEPSC regulation. However, future experiments are required to determine if deletion of multiple PLC isoforms impacts the [Ca^2+^]_*o*_-dependent regulation of spontaneous release.

We further investigated the role of PLC activity in synaptic transmission by eliciting exocytosis of the RRP of synaptic vesicles with hypertonic sucrose solution during PLC inhibition. PLC inhibition with U73122 produced a substantial decrease in the RRP that was partially rescued by application of PDBu. If the same mechanism accounted for the [Ca^2+^]_o_ -dependent increases in mEPSC frequency observed here and reported previously, this would suggest CaSR and other GPCRs stimulate spontaneous transmission (Vyleta and Smith, 2011) by increasing the RRP. RRP size regulation alone does not explain why CaSR activation also decreases evoked transmission (Phillips et al., 2008), requiring more than one effect of the CaSR and distinct vesicle pools for evoked and spontaneous neurotransmission. However, it has been suggested that U73122 elicits some portion of its inhibition of PLC activity by interaction with PIP_2_, rather than by solely inhibiting the PLC enzyme itself (Burgdorf et al., 2010). Given that PIP_2_ is linked to both exo- and endocytosis (Sun et al., 2007), this suggests the possibility that PIP_2_ function is impaired under U73122-treatment leading to some of the effects on spontaneous release and RRP replenishment seen.

Using chromaffin cells, Walter et al. (2017) also determined that phorbol ester is not a strong mediator in exocytosis. They found that increasing intracellular PIP_2_ increased exocytosis, however, increased intracellular DAG did not (Walter et al., 2017). Contrasting with our findings, however, this paper also found that PLC inhibition with U73122 enhanced RRP replenishment due to the inhibition of PIP_2_ degradation and therefore maintenance of a high concentration of this molecule, though this effect was small. Whether this discrepancy on the role of PLC inhibition on RRP replenishment is due to differences in the RRP probing methods (hypertonic sucrose versus increased [Ca]_i_), different cell types, or some difference in the detection of events (postsynaptic whole-cell patch which receives input from many neurons versus membrane capacitance of a single cell), it does highlight a role for PIP_2_ as another functional effector, rather than solely as a precursor to IP_3_ and DAG.

### Study limitations

U73343 does not inhibit PLC but its stimulatory action on mEPSC frequency shows that it is not inactive (Figures 2B, C). U73122 also increased spontaneous neurotransmission transiently, supporting the idea that this stimulation is not PLC-mediated but reflects an off-target effect. We attribute differences between the two agents to inhibition of PLC but, as mentioned, use an appropriate incubation period such that the off-target effects of U73122 should no longer be in effect and PLC inhibition by U73122 should be complete.

### Conclusion

Our data indicate that PLC activity is necessary to enhance the spontaneous release of glutamate in response to elevated [Ca^2+^]_o_ in neocortical neurons. We propose that this occurs, at least in part, due to an increase in the size of the RRP which increases the overall likelihood of spontaneous release.

## Data availability statement

The raw data supporting the conclusions of this article will be made available by the authors, without undue reservation.

## Ethics statement

All animal procedures were approved by VAPORHCS IACUC in accordance with the U.S. Public Health Service Policy on Humane Care and Use of Laboratory Animals and the NIH.

## Author contributions

MF, NV, and SS contributed to the writing of the manuscript, participated in research design, and performed data analysis. MF and NV conducted experiments. All authors contributed to the article and approved the submitted version.
